# Transient Anisocoria after Corneal Collagen Cross-Linking

**DOI:** 10.1155/2014/487860

**Published:** 2014-09-08

**Authors:** George D. Kymionis, Michael A. Grentzelos, Nela Stojanovic, Theodore A. Paraskevopoulos, Efstathios T. Detorakis

**Affiliations:** ^1^Vardinoyiannion Eye Institute of Crete (VEIC), Faculty of Medicine, University of Crete, Heraklion, 71003 Crete, Greece; ^2^Bascom Palmer Eye Institute, Miller School of Medicine, University of Miami, Miami, FL 33136, USA

## Abstract

*Purpose*. To report a case with transient anisocoria after corneal collagen cross-linking (CXL). *Methods*. Case report. *Results*. A 24-year-old male underwent corneal collagen cross-linking (CXL) in his right eye for keratoconus. At the end of the procedure, the pupil of the treated eye was irregular and dilated, while the pupil of the fellow eye was round, regular, and reactive (anisocoria). The following day, pupils were round, regular, and reactive in both eyes. *Conclusion*. Anisocoria may be a transient and innocuous complication after CXL. A possible cause for this complication might be the anesthetic drops used before and during the surgical procedure or/and the ultraviolet A irradiation during the treatment.

## 1. Introduction

Corneal collagen cross-linking (CXL) is a minimally invasive surgical procedure used for the management of corneal ectatic disorders such as keratoconus and post-LASIK corneal ectasia [1, 2]. Riboflavin in conjunction with ultraviolet A (UVA) irradiation strengthens the corneal tissue and increases biomechanical stability of the ectatic cornea [1]. Several studies have reported stabilization and arresting keratoconus progression after CXL treatment [1–6]. However, several complications after CXL have already been reported [7–14]. In this case, we present a patient with dilated and irregular pupil of the CXL-treated eye at the end of the procedure.

## 2. Case Report

A twenty-four-year-old male with progressive keratoconus presented to our institute for evaluation. At the time of examination, uncorrected distance visual acuity (UDVA) was 20/100 in the right eye and 20/200 in the left eye. Corrected distance visual acuity (CDVA) was 20/25 (manifest refraction +0.25–3.50 × 70) and 20/40 (manifest refraction +1.50–6.50 × 110) in the right and left eyes, respectively. Keratometric readings were 47.79/44.29 diopters (D) and 52.81/46.56 D in the right and left eyes, respectively. Central corneal thickness (CCT) was 451 *μ*m and 429 *μ*m for the right and left eyes, respectively. Ocular and medical histories were unremarkable. Slit-lamp examination showed no other anterior or posterior abnormality. The patient was advised to undergo CXL treatment in both eyes starting with the right eye. The patient underwent CXL treatment in his right eye.

**Figure 1:**
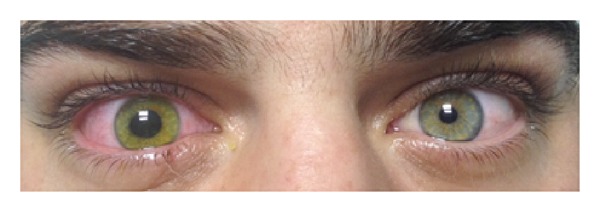
Patient's photo showing irregular and dilated pupil of the treated right eye at the end of the CXL procedure (anisocoria).

After topical anesthesia with proparacaine hydrochloride 0.5% eye drops (Alcaine; Alcon Laboratories, Inc., Fort Worth, Texas, USA), CXL was conducted according to the Dresden protocol [1]. At the end of the procedure, the pupil was irregular and dilated but reactive to light; the pupil of the fellow eye was round, regular, and reactive (anisocoria) (Figure 1). A bandage contact lens (BCL) was applied until full reepithelialization. Intraocular pressure estimated with digital palpation through closed eyelids was normal and equal compared to the contralateral eye. Postoperative medication included ofloxacin (Exocin, Allergan Pharmaceuticals Ltd.) four times daily and chloramphenicol/dexamethasone drops (Dispersadron, Thea Laboratories, Inc.) four times daily until the epithelium healed completely. The patient was encouraged to use artificial tears at least six times per day.

**Figure 2:**
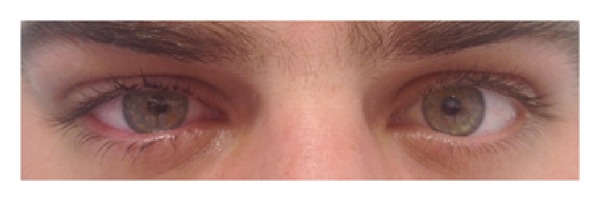
Patient's photo with round and regular pupil of both eyes one day after CXL treatment of the right eye.

On the first postoperative day, slit-lamp examination of the treated eye revealed a regular, round, and reactive pupil (Figure 2); cornea showed slight edema and epithelial defect. On the fourth postoperative day, corneal epithelium was completely healed and the contact lens was removed.

## 3. Discussion

CXL is generally considered a safe and efficient surgical intervention for the treatment of corneal ectatic disorders, such as keratoconus and post-LASIK ectasia [1–6]. Nevertheless, complications may occur after CXL and have already been reported [7–14]. Although some of them are mild and innocuous, others could be severe and result in permanent decrease of visual acuity.

In this case report, we describe a patient with irregular and dilated pupil of the treated eye immediately after CXL procedure; the pupil of the fellow eye was round, regular, and reactive (anisocoria). The patient had no history of any iris or pupil size, shape, or reaction anomaly. Although this complication was mild, transient, and innocuous, it raises questions regarding its cause. One of the possible explanations may be the effect of proparacaine hydrochloride ophthalmic solution, which was used for anesthesia before and during the procedure; one of the proparacaine hydrochloride eye drops' side effects may be pupillary dilation or cycloplegia. Ultraviolet A irradiation during the treatment might be another possible cause for this complication, even though the possible mechanism of the ultraviolet A irradiation effect on the pupil size remains unclear. Whatever the cause, the post-CXL anisocoria was innocuous. Even though there is no evident cause for this transient and innocuous complication after CXL, we believe that it is important to report and differentiate it from any preexisting pupillary disorder.
